# Association between relative handgrip strength and hypertension in Chinese adults: An analysis of four successive national surveys with 712,442 individuals (2000-2014)

**DOI:** 10.1371/journal.pone.0258763

**Published:** 2021-10-28

**Authors:** Qiang Feng, Chongmin Jiang, Mei Wang, Rui Cai, Huan Wang, Dongming Wu, Fubaihui Wang, Lizi Lin, George P. Nassis

**Affiliations:** 1 Department of Maternal and Child Health, School of Public Health, Peking University, Beijing, China; 2 Department of Fitness Surveillance Centre, China Institute of Sport Science, Beijing, China; 3 Department of Occupational and Environmental Health, School of Public Health, Sun Yat-Sen University, Guangzhou, China; 4 Physical Education Department–College of Education (CEDU), United Arab Emirates University, Al Ain, Abu Dhabi, United Arab Emirates; 5 Department of Sports Science and Clinical Biomechanics, SDU Sport and Health Sciences Cluster, University of Southern Denmark, Odense, Denmark; University of Hong Kong, HONG KONG

## Abstract

**Objectives:**

This study aimed to identify the 15-year trends of handgrip strength-to-weight (relative HS) and assess the association between relative HS and hypertension among Chinese adults aged 20–69.

**Methods:**

Using a complex, stratified, multistage probability cluster sampling, we analyzed data collected from 712,442 adults aged 20–69 years in four successive national surveys (2000–2014). We used a handheld dynamometer to measure strength and divided by body weight to calculate the relative HS. Blood pressure was recorded with a sphygmomanometer and hypertension was defined as resting systolic blood pressure at least 140 mmHg or diastolic at least 90 mmHg. The Mann-Kendall trend test examined trends in relative HS over time. We also computed odd ratios (OR) and 95% confidence intervals (95% CI) by tertile of relative HS and examined the association between relative HS and hypertension.

**Results:**

The relative HS level decreased with the increase of age in both male and females (*p*_trend_ <0.001). In each of four surveys, one interquartile decrease in relative HS was associated with an increased risk of hypertension by 44% (OR = 1.44, 95%CI: 1.40–1.47), 58% (OR = 1.58, 95%CI: 1.54–1.62), 48% (OR = 1.48, 95%CI: 1.45–1.52), 43% (OR = 1.43, 95%CI: 1.40–1.47), respectively.

**Conclusion:**

In the Chinese adult population, the relative HS level decreased from 2000 to 2014 across all ages in both males and females. A lower relative HS was associated with a higher risk of hypertension. The findings provided evidence for the association between muscle strength and hypertension in large-scaled population.

## Introduction

Hypertension is a common health problem worldwide [1]. The China Patient-Centered Evaluative Assessment of Cardiac Events (PEACE) study indicated that nearly half of Chinese adults aged 35–75 years old had hypertension, and fewer than one in twelve were in control of their blood pressure [2]. The poor blood pressure control warrants greater efforts on primary prevention in China [2, 3].

Regular exercise is an essential component of a lifestyle intervention for the primary prevention of hypertension [4]. Absolute handgrip strength (HS) is commonly used as a surrogate measurement of overall muscle strength, which can be developed by regular exercise [5, 6]. There is global variation in absolute HS across different countries and regions, with the HS level being higher in Western countries than in Asian countries [7]. In a previous study, we have observed a decreasing absolute HS with rising age in both sexes among the Chinese population during the past 15 years [8].

The evidence regarding the association between absolute HS and hypertension is inconclusive. A meta-analysis summarized that an increase of absolute HS exercise was associated with a lower risk of hypertension in adults, but these findings had limitations given the small number of low-quality randomized controlled trials [9]. Two cross-sectional studies conducted in Western countries (the Netherlands and the USA) indicated that increased absolute HS was associated with higher blood pressure in the general population [10, 11]. We found only one relevant study conducted in rural areas in China, reporting that increased absolute HS was associated with a higher risk of hypertension in women [12].

The above studies had one common feature: they used absolute HS, rather than relative HS (handgrip strength to body weight or BMI ratio), which might be more comparable among people with different body mass [13, 14]. When using the relative HS, three studies conducted in Asian countries (Korea and China) indicated that a higher relative HS was associated with a lower blood pressure, especially in the adult men [13–15]. However, the sample sizes were limited in the previous studies with the numbers of participants ranging from 927 to 5,014.

In the present study, we analyzed the Chinese National Survey on Adults’ Fitness in 2000, 2005, 2010, and 2014. The study aimed to identify the 15-year trends of relative HS in different age and sex groups from 2000 to 2014 and assess the association between relative HS and hypertension among Chinese adults aged 20–69.

## Methods

### Study design and participants

We used the data from 2000, 2005, 2010, and 2014 Chinese National Survey on Adults’ Fitness, the largest nationally representative survey of civilians in China, using a complex, stratified, multistage probability cluster sampling design. The detail of the recruitment has been described elsewhere [8].

In brief, the 31 provinces, autonomous regions, and municipalities in mainland China were covered in the first stage. At the second stage, subject to their economic positions weighted by GDP assessment, three sub-provincial or prefectural-level cities (i.e., a division ranked between province and county in China’s administrative structure) were randomly selected from each province. At the third stage, three urban districts (or three rural counties) of each city were picked out. At the fourth stage, three city streets (or rural towns) were chosen. At the fifth stage, two street community societies (or villages) were selected. At the final stage, at each street community society (or village), eligible participants living there for more than three years were selected by systematic sampling method.

The study protocol had approval from the ethical review committee of the China Institute of Sport Science. Each enrolled participant signed an informed consent form.

### Procedures

Through the questionnaire survey and standard physical and physiological measurement, the trained investigators collected the data. We investigated career, education, urban and rural place, nationality (Han or minority) with the questionnaire. For exercise time, the participants were asked to recall the number of days and the exercise duration in the usual past week during the last year. The questionnaire was validated among Chinese adults (n = 2014, aged 20–75 years) [8].

Participants’ weight and height were measured using a calibrated digital scale, stadiometer (Jianmin II, China), Before the measurement, participants with lightweight clothes took off their shoes. The measured weight was rounded up to 0.1kg and height was rounded up to 0.1cm, respectively. The blood pressure measurement was conducted by the auscultatory method by qualified medical staff. The Riva-Rocci sphygmomanometer, and a medical stethoscope (Yuwell, China) were used for the measurement. The systolic pressure was defined as the first sound heard after releasing the occluded artery. The diastolic pressure corresponded to the final sound heard [16]. Systolic and diastolic blood pressures were recorded in mmHg.

The following measures were taken before and during the data collection:

1. The subjects were asked not to engage in vigorous physical activity within 1–2 hours before the test.
2. The subjects were asked to sit on the chair in silence for 10–15 minutes before the test, and relax.
3. The mercury column of the sphygmomanometer was checked before measurement. If the column was not at the “0” position, the position was corrected. Furthermore, the mercury column was inspected for the presence of bubbles, and if there were bubbles, it was not used.
4. When measuring blood pressure, it was ensured that the upper arm was not pressed tightly by the sleeve of the jacket. It was ensured that the lower edge of the cuff was located at least 2.5 cm above the cubital fossa.
5. When re-testing was needed (the first test was failed by some reasons), it was ensured that the mercury column of the sphygmomanometer dropped to zero before re-testing. Blood pressure re-testing was carried out 10–15 minutes rest on chair after the initial test. Hypertension was defined as resting systolic blood pressure at least 140 mmHg or diastolic at least 90 mmHg according to the common standard [17].

### Handgrip measurement

We used the handgrip meter (Jianmin II, China) to measure the dominant hand handgrip strength. Before the measurement, the subjects held the grip handles with dominant hand and adjusted the grip width to the appropriate force grip. During the measurement, the participants’ body was upright, feet were naturally separated at shoulder width, while arms were inclined and drooping. After three consecutive tests, with adequate rest interval in between, we recorded each participant’s best score as the final result. The grip strength test value is in kg, rounded up to 0.1kg.

### Body fat measurement

We used the Skinfold Thickness Meter (Jianmin II, China) to measure the skinfold thickness from three different body sites (triceps, subscapular, and abdomen). We measured three times for each body position and took the median value or two of the same value as the final result. We added three different skinfold thickness measurements to reflect the body fat (skinfold thickness).

### Statistical analyses

We described the median (interquartile, IQR) of relative HS (HS to body weight ratio) in different survey years since relative HS was not normally distributed. In the study, the bootstrap percentile method (the number of bootstrap replicates is 500 times) was used to calculate confidence intervals for medians [18, 19]. We used the Mann–Whitney U test or Kruskal–Wallis test to detect the difference in relative HS between sex, urban-rural, nationality, inner-province socio-economic status, education levels, career, nutritional status in the same survey year. We performed the Mann-Kendall trend test to examine the relative HS trend and survey years or increase age groups [20].

To explore the relationship between relative HS and hypertension, we used the tertiles to separate the subjects into three groups (low relative HS, middle relative HS, and high relative HS) according to the different sex, survey year, and age group, then calculated the prevalences of hypertension in different relative HS groups. The Mann-Kendall trend test was used to examine the trend of hypertension prevalences in different years among different tertiles of relative HS.

We used the general linear mixed model to estimate the effect value and odd ratios. Relative HS was analyzed both as a continuous variable and a categorical variable (tertiles, with the highest group as the reference group). We presented the results of two statistical models, in which the province of each participant was used as the random effect. We only put exposure and outcome varibales in the crude model. In model 1, we adjusted with age and sex as covariates; In model 2, we further adjusted for the region (rural or urban), the inner-province socio-economic status (low, middle, high), the nationality (Han or minority, data available in three surveys in 2005, 2010 and 2014), the education level (data available in three surveys in 2005, 2010 and 2014), career(government official, technical staff, office staff, commercial and service, agriculture, forestry, animal husbandry and fishery, production, and transportation, soldier, other), and exercise level (whether taking exercise for at least 60 minutes in the usual past week) as covariates.

In the sensitivity analyses [21, 22], we additionally adjusted for the sum of skinfold thickness based on the adjusted model 2. Besides, we calculated another index (HS to BMI ratio) to reflect relative HS and used the same models to analyze its association with hypertension. We did the stratified analysis among underweight, normal, overweight and obesity groups using the body mass index for the category(below18.5 kg/m^2^ for underweight group, between 18.5 kg/m^2^ and 23.9 kg/m^2^ for normal group, above 24.0 kg/m^2^ for overweight and obesity group) [23].

All the statistical analyses were performed with R software, version 3.6.1. A two-sided *p*-value <0.05 was considered as statistical significance.

## Results

We included 712,442 participants (177,517 in 2000; 188,521 in 2005; 179,870 in 2010; 166,534 in 2014), while 8,838 participants were excluded for missing main outcome and exposures. The general characteristics and HS levels of the study population in each of the four national surveys were shown in Table 1. Both absolute HS and relative HS had a decreasing trend across the different survey years.

**Table 1:** The general characteristic and handgrip strength levels of the study population in each of the four national surveys from 2000 to 2014.

	2000	2005	2010	2014
Sample Size	177,517	188,521	179,870	166,534
sex				
male	89,340 (50.3%)	94,002 (49.9%)	90,350 (50.2%)	83,184 (50.0%)
female	88,177 (49.7%)	94,519 (50.1%)	89,520 (49.8%)	83,350 (50.0%)
Regions				
Rural	61,977 (34.9%)	67,185 (35.6%)	63,739 (35.4%)	58,130 (34.9%)
Urban	115,540 (65.1%)	121,336 (64.4%)	116,131 (64.6%)	108,404 (65.1%)
Age group				
20–24	19,330(10.9%)	20,306(10.8%)	19,407(10.8%)	18,726(11.2%)
25–29	19,262(10.9%)	20,292(10.8%)	19,210(10.7%)	18,768(11.3%)
30–34	19,191(10.8%)	20,582(10.9%)	19,362(10.8%)	18,697(11.2%)
35–39	19,126(10.8%)	20,214(10.7%)	19,265(10.7%)	18,419(11.1%)
40–44	19,209(10.8%)	20,696(11.0%)	19,358(10.8%)	17,486(10.5%)
45–49	18,927(10.7%)	20,038(10.6%)	19,298(10.7%)	17,217(10.3%)
50–54	18,701(10.5%)	20,022(10.6%)	19,328(10.7%)	16,988(10.2%)
55–59	17,910(10.1%)	19,460(10.3%)	19,156(10.6%)	16,380(9.8%)
60–64	13,129(7.4%)	13,740(7.3%)	12,813(7.1%)	12,279(7.4%)
65–69	12,732(7.2%)	13,171(7.0%)	12,673(7.0%)	11,574(6.9%)
Inner-province socio-economic status				
capital city	63,121(35.6%)*	64,819(34.4%)	58,972(32.8%)	55,584(33.4%)
fair economy	54,084(30.5%)	63,087(33.5%)	63,053(35.1%)	55,671(33.4%)
low economy	51,810(29.2%)	60,615(32.2%)	57,845(32.2%)	55,279(33.2%)
Education				
no formal education	NA^#^	14,363(7.6%)	10,368(5.8%)	7,558(4.5%)
primary school		25,258(13.4%)	21,499(12.0%)	17,694(10.6%)
junior high		48,865(25.9%)	47,319(26.3%)	40,849(24.5%)
senior high		49,426(26.2%)	47,156(26.2%)	41,414(24.9%)
University or above		50,596(26.8%)	53,521(29.8%)	58,859(35.3%)
Career				
government official	13,218(7.4%)	15,385(8.2%)	18,011(10.0%)	9,365(5.6%)
technical staff	25,294(14.2%)	28,766(15.3%)	27,144(15.1%)	25,269(15.2%)
officer staff	23,233(13.1%)	20,771(11.0%)	22,409(12.5%)	25,647(15.4%)
Commercial and service	11,235(6.3%)	12,315(6.5%)	14,890(8.3%)	17,005(10.2%)
Agriculture, forestry, animal husbandry and fishery	55,576(31.3%)	53,137(28.2%)	39,204(21.8%)	31,143(18.7%)
Production and transportation	38,739(21.8%)	32,945(17.5%)	28,440(15.8%)	26,284(15.8%)
soldier	NA^#^	357(0.2%)	669(0.4%)	272(0.2%)
other(including no occupation)	9,885(5.6%)	24,824(13.2%)	29,083(16.2%)	28,794(17.3%)
Nation				
Han	NA^#^	167,193(88.7%)	157,437(87.5%)	145,031(87.1%)
Minority		21,328(11.3%)	22,433(12.5%)	21,484(12.9%)
Nutritional status				
height	162.4(8.1)	162.5(8.3)	162.8(8.4)	163.5(8.4)
weight	61.4(10.6)	61.7(11.1)	63(11.4)	63.8(11.6)
BMI(kg/m2)	23.2(3.2)	23.3(3.4)	23.7(3.4)	23.8(3.4)
skinfold thickness^&^	57.2(23.7)	58.1(24.5)	56.2(22.3)	59.8(21.9)
Thinness	9,085(5.1%)	9,847(5.2%)	7,593(4.2%)	6,530(3.9%)
Normal weight	101,487(57.2%)	105,765(56.1%)	93,973(52.2%)	85,717(51.5%)
overweight	53,111(29.9%)	56,864(30.2%)	59,666(33.2%)	55,889(33.6%)
obese	13,834(7.8%)	15,969(8.5%)	18,508(10.3%)	18,007(10.2%)
Absolute HS^$^	34.3(18.1)	33.5(18.2)	33.0(18.2)	32.3(17.8)
Relative HS^$^	0.577(0.223)	0.563(0.219)	0.543(0.211)	0.526(0205)

* In year 2000, 8502(4.79%) participants are missing Inner-province socio-economic status data.

^#^ In year 2000, no investigation on Education, Nation and soldier career.

^$^ Present the median (interquartile, IQR), since variables are not normally distributed.

^&^ Add triceps, subscapular, and abdomen skinfold thickness together.

**Fig 1:**
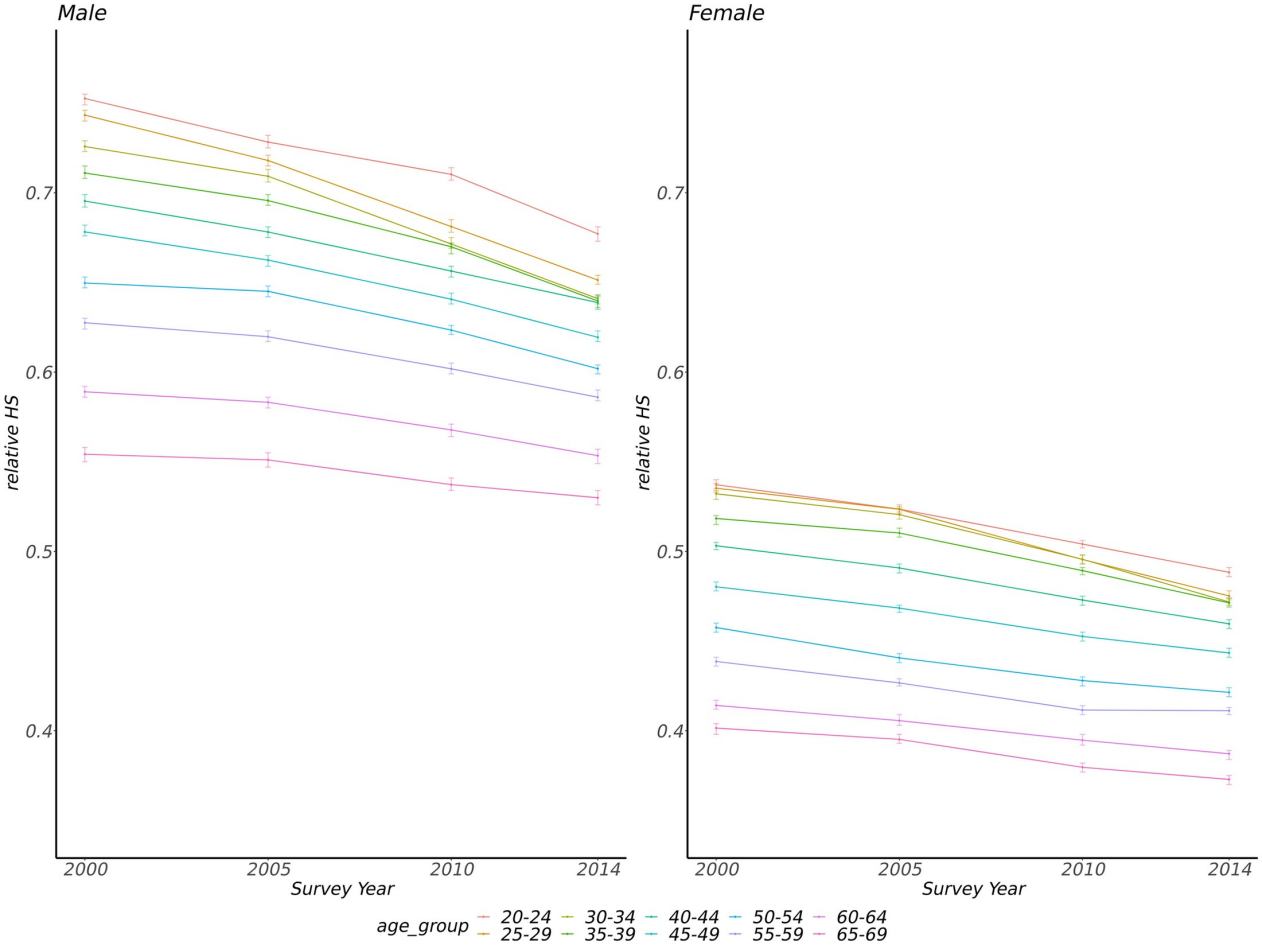
The medians with 95% confidence intervals of relative handgrip strength among different age groups in four survey years (error bars represent 95% confidence intervals for medians).

**Fig 2:**
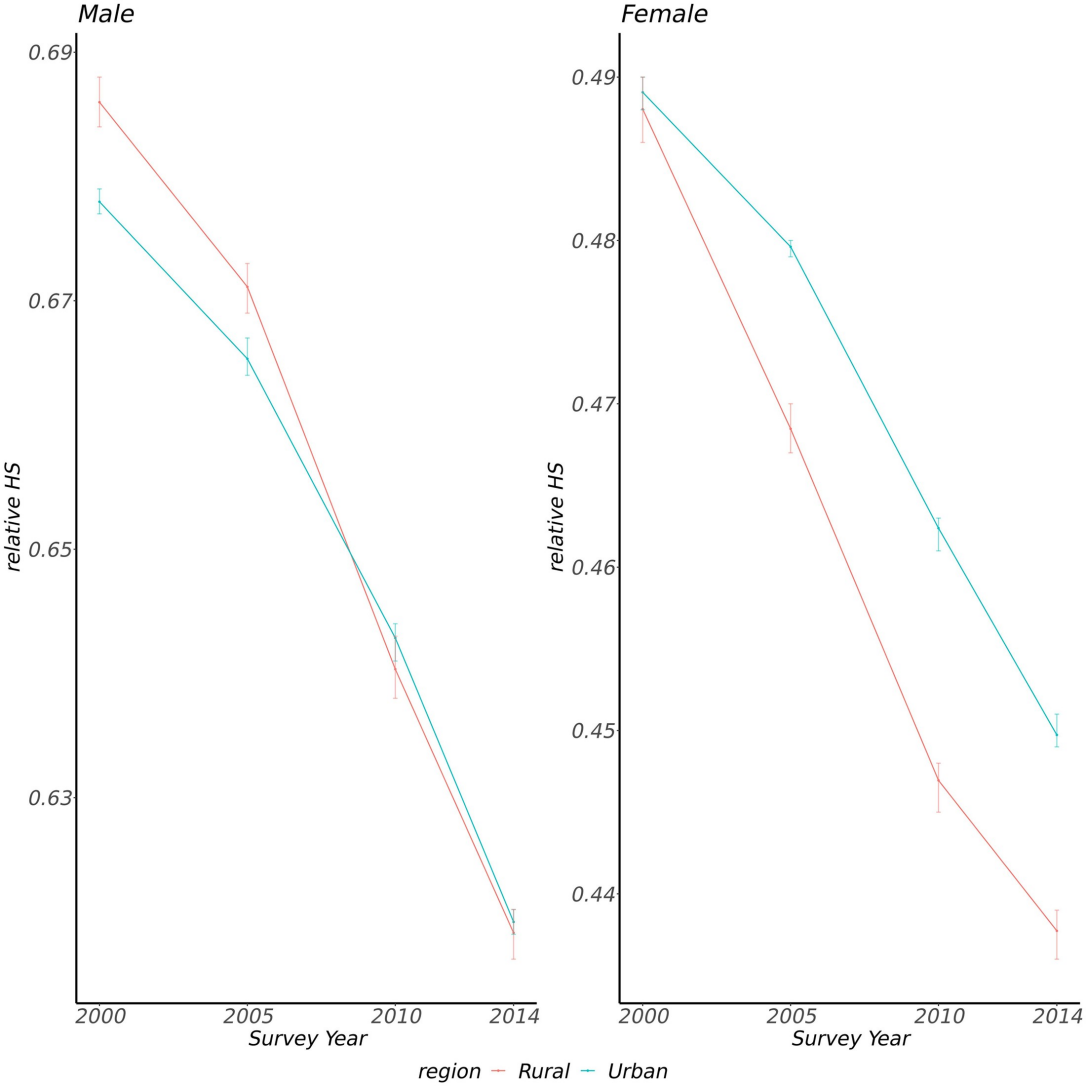
The medians with 95% confidence intervals of relative HS in rural or urban areas in four survey years (error bars represent 95% confidence intervals for medians).

The median of relative HS decreased across all age groups in both men and women from 2000 to 2014 (all *p* trend <0.001, Fig 1, S1 Table). The relative HS median was higher in men than in women across different survey years (all *p* <0.001). It also decreased in urban and rural areas from 2000 to 2014 (all *p* trend <0.001, Fig 2). The median of relative HS in women was higher in urban areas than those in rural areas across different survey years except for the year 2000 (*p*<0.001 for 2005, 2010, and 2014; *p* = 0.828 for the year 2000, S2 Table). Men who lived in urban areas had a lower median of relative HS than those who lived in rural areas in the year 2000 and 2005 (*p*<0.001, S2 Table), while the median of the male relative HS was higher in urban areas than those in rural areas in the year of 2010 (*p* <0.001, S2 Table), and there was no difference in the year of 2014 (*p* = 0.079, S2 Table).

**Fig 3:**
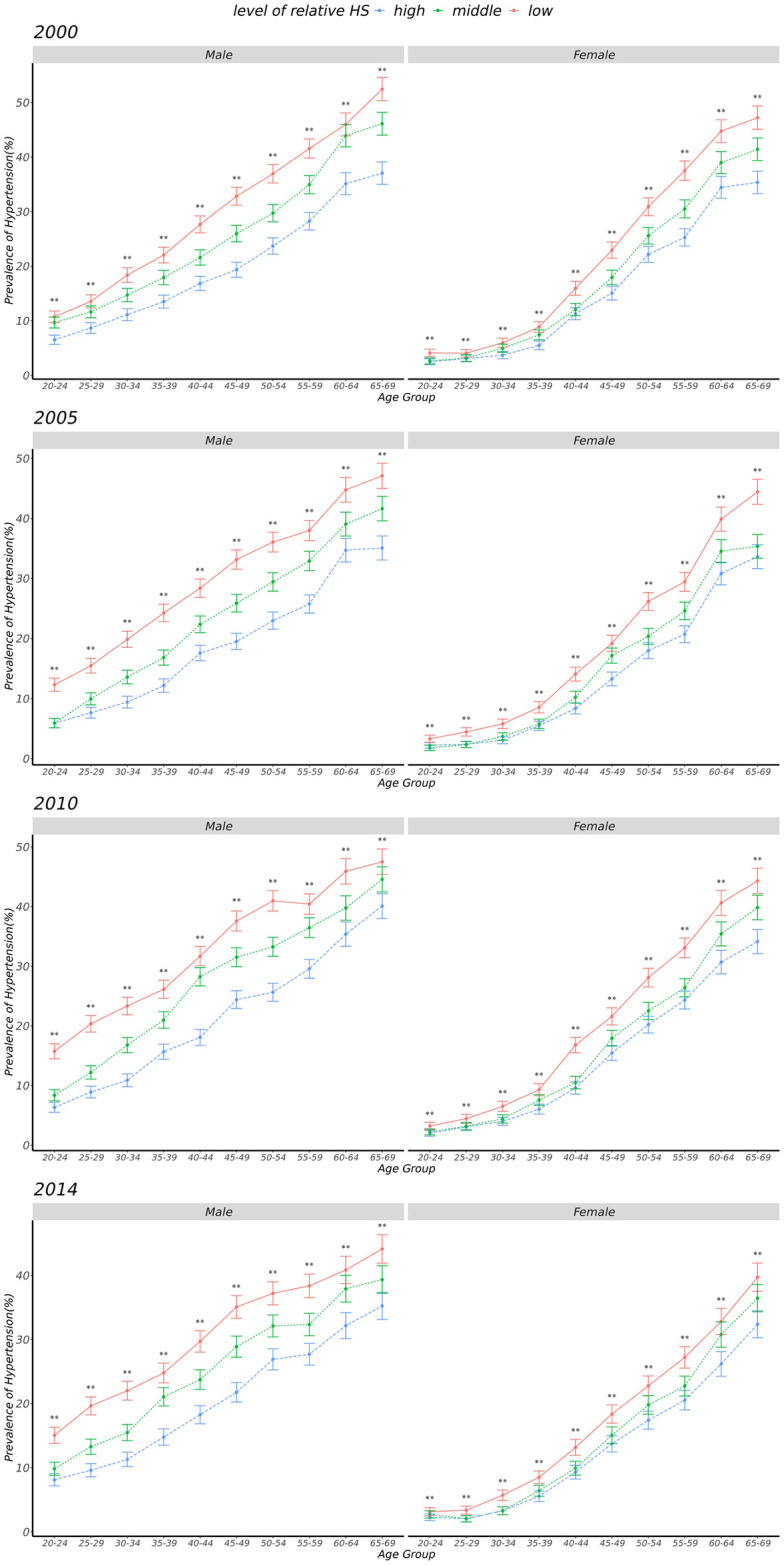
The prevalence of hypertension among three tertile groups of handgrip strength across different age groups in four survey years (error bars represent 95% confidence intervals for medians, **represent *p*<0.001).

As shown in Fig 3 (details in S3 Table), the high relative HS groups had the lowest prevalence of hypertension in both sexes across different survey years (*p*<0.001). In addition, the prevalence of hypertension increased with increasing age groups (*p* trend <0.001) and was higher in men than in women in different survey years (all *p* <0.05, details in S4 Table).

As shown in Table 2, the higher relative HS was consistently associated with a lower risk of hypertension in different survey years in the adjusted models. Compared with the high relative HS group, the middle and low relative HS groups had a higher risk of hypertension in different survey years ranging from 1.26 to 1.31 and from 1.58 to 1.72, respectively. Using the continuous variable of relative HS, we also found an association between relative HS and the risk of hypertension in different survey years (Table 3). One interquartile range (IQR) deviation decrease in relative HS was associated with higher risk of hypertension in different survey years (OR = 1.44, 95%CI:1.40–1.47 for 2000; OR = 1.58, 95%CI:1.54–1.62 for 2005; OR = 1.48, 95%CI:1.45–1.52 for 2010; OR = 1.43, 95%CI:1.40–1.47 for 2014).

**Table 2:** The associations between relative HS (category variable) and hypertension.

	High HS	Middle HS		Low HS	
		OR (95% CI)	*p*	OR (95% CI)	*p*
2000					
Crude	REF	1.26(1.22–1.30)	<0.001	1.53(1.48–1.57)	<0.001
Model 1	REF	1.28 (1.24–1.32)	<0.001	1.58 (1.54–1.63)	<0.001
Model 2	REF	1.31 (1.26–1.35)	<0.001	1.61 (1.56–1.66)	<0.001
2005					
Crude	REF	1.23(1.20–1.27)	<0.001	1.64 (1.59–1.69)	<0.001
Model 1	REF	1.26 (1.22–1.30)	<0.001	1.71 (1.66–1.77)	<0.001
Model 2	REF	1.27 (1.23–1.31)	<0.001	1.72 (1.67–1.77)	<0.001
2010					
Crude	REF	1.25(1.22–1.29)	<0.001	1.59(1.55–1.64)	<0.001
Model 1	REF	1.27 (1.24–1.31)	<0.001	1.65 (1.61–1.71)	<0.001
Model 2	REF	1.28 (1.24–1.32)	<0.001	1.65 (1.60–1.70)	<0.001
2014					
Crude	REF	1.23(1.19–1.27)	<0.001	1.53(1.48–1.58)	<0.001
Model 1	REF	1.24 (1.20–1.28)	<0.001	1.58 (1.53–1.63)	<0.001
Model 2	REF	1.26 (1.22–1.30)	<0.001	1.58 (1.53–1.64)	<0.001

Notes: HS = handgrip strength; OR = odds ratio; CI = confidence interval; REF = reference group.

Crude Model: with the province of each participant was used as the random effect.

Model 1: adjusted for age and sex.

Model 2: adjusted for age, sex, region (urban or rural), inner-province economic status (high, middle, low), nationality, education level, career, exercise (at least 60 mins/week or not).

**Table 3:** The associations between relative HS (continuous variable) and hypertension (per IQR decrease).

	OR (95%CI)	*p*
2000		
Crude	1.45(1.42–1.47)	<0.001
Model 1	1.43 (1.39–1.46)	<0.001
Model 2	1.44(1.40–1.47)	<0.001
2005		
Crude	1.38 (1.35–1.40)	
Model 1	1.58 (1.54–1.62)	<0.001
Model 2	1.58(1.54–1.62)	<0.001
2010		
Crude	1.26(1.24–1.28)	<0.001
Model 1	1.49 (1.46–1.53)	<0.001
Model 2	1.48 (1.45–1.52)	<0.001
2014		
Crude	1.15(1.13–1.17)	<0.001
Model 1	1.43 (1.40–1.47)	<0.001
Model 2	1.43 (1.40–1.47)	<0.001

Notes: HS = handgrip strength; IQR = interquartile; OR = odds ratio; CI = confidence interval.

Crude Model: with the province of each participant was used as the random effect.

Model 1: adjusted for age and sex.

Model 2: adjusted for age, sex, region (urban or rural), inner-province economic status (low, middle, high), nationality, education level, career, exercise (at least 60 mins/week or not).

All the sensitivity analyses showed results similar to our main results. We further adjusted the skinfold thickness based on Model 2. The odd ratios of middle and low tertiles for hypertension were 1.10–1.16 and 1.26–1.34, respectively (all *p*<0.0001, S5 Table). Furthermore, another sensitive analysis showed that HS to BMI ratio was also associated with hypertension, which was directionally consistent with the main results (S6 and S7 Tables). In the stratified analysis, the odd ratios of middle and low tertiles for hypertension in model 2 were 1.04–1.07 and 1.04–1.12 in normal weight group, respectively. And the odd ratios of middle and low tertiles for hypertension in model 2 were 1.02–1.08 and 1.18–1.24 in overweight and obesity group (S8 Table). We also separate different sex (S9 and S10 Tables) and age groups (S11 Table) to do the subgroup analysis. The result is correspondence with the main result.

## Discussion

This study found that the relative HS was negatively associated with hypertension in national surveys data for Chinese adult population. One interquartile decrease in relative HS was associated with an increased risk of hypertension in the range of 43% to 58%. This finding was based on a big number of individuals (712,442 adults) and this is a strength of our study. Finally, relative HS significantly decreased during the past 15 years in both men and women in China. A similar trend was seen in different age groups, in both urban and rural areas.

The finding of a 43–58% reduction of risk in hypertension with one interquartile decrease in relative HS is of public health significance. High blood pressure is the leading modifiable risk factor for cardiovascular disease and premature death worldwide [24]. In our data, one interquartile change of HS is translated to 0.15 to 0.17 for males and 0.12 to 0.14 for females among different age groups. In the 60–69 age group of the year 2014, if a man with 60kg bodyweight could improve his absolute handgrip strength by 5.02kg or a woman with 50kg bodyweight could improve his absolute handgrip strength 2.20kg, their reduction of risk in hypertension would decrease 43%. This improvement of muscle strength can be achievable for a person by doing exercise [25, 26].

The decline of HS, which may reflect the change in whole body strength [6], has been found to have detrimental effects on health, including sarcopenia in the elderly [27], chronic non-communicable diseases [28], and have a higher risk of all-cause mortality [29]. The increased risk of hypertension in the adult population with lower relative HS found in our study is consistent with the results from the Korean Longitudinal Study on Health and Aging, in which relative HS was inversely associated with risk of hypertension in adults [15, 30]. However, several studies that used absolute HS had found different results. Taekema et al [10] measured blood pressure and handgrip strength in 670 middle-aged adults and 550 older people. They found that a high blood pressure was associated with higher HS among the elderly. Samely, in children and adolescents, the absolute HS is also found to be positive with blood pressure among participants [31, 32]. The conflicting results may be due to the difference between absolute and relative HS. It was found that body height and weight were independently associated with HS [33]. Some studies reported that the relative value of HS had an advantage for comparing different weight groups [14, 34, 35]. Chatterjee et al [36] found that HS was positively correlated with body weight(r = 0.86 to 0.87, both hands, aged from 7–73 years). So, we make reasonable inferences that the relative HS would be more comparable at the population level.

Our findings suggested that relative HS is inversely associated with the risk of hypertension. Furthermore, in the sensitive analysis, stratified BMI groups’ analysis showed that the body mass index did not affect the negative association between relative HS and hypertension, which was in agreement with a rescent study [37]. One possible explanation of strength level associated with hypertension is that people who participate in physical activities more frequently have higher relative HS. The strength-related activities could cause shear stress on vessels in the whole body, which might raise the level of nitric oxide synthase and endothelium-derived nitric oxide [38–40].

We found that relative HS has been decreasing among Chinese male and female adults in both urban and rural areas since 2000. The results extended findings from the previous study, which reported the decreasing trend in absolute HS in these successive national surveys [8]. In recent years, there has been a decreasing trend in occupational, physical activity, domestic physical activity, and leisure time activity in China [8, 41], which was probably associated with the decrease of relative HS [42, 43]. To our knowledge, this is the first study which provides HS reference values for Asian population based on a very big representative sample. Accordingly this data could be used as reference values, for future comparisons [44].

This is the first national study to display the difference of relative HS between male and female in China, and its result, higher relative HS in male adult was consistent with the studies of relative HS in other populations [45, 46]. Moreover, the urban-rural disparity should be paid attention to. Men and women living in the rural area seemed to have lower relative HS than those in urban areas. The possible explanation could be that there was less manual work with progressive years than before in the rural area, especially with regards to the female population. In contrast, the fast changing environment reinforcing physical activity and training in urban cities, like the building of exercise facilities, may explain the higher strength level reported for the urban population in our study [47, 48].

This study had notable strengths, identifying epidemiological characteristics of relative HS with a sizeable nationally-representative sample size in China and repeated measures during the past 15 years. However, the study had several limitations that should be noted. First, the cross-sectional nature of the national representative data precluded causal inference. Longitudinal studies should be conducted to demonstrate the relationship between relative HS and hypertension using a stronger experimental design. Second, we did not collect hypertension-related risk factors such as energy intake, sodium and potassium intake. It was infeasible to collect comprehensive diet information in large-scale national surveys. However, the consistent association results was found in different survey years and stratified analyses by gender, urban / rural, normal-weight /overweight or obese, indicating the potential bias due to unadjusted factors might be minor. Third, we used body weight and BMI to calculate relative HS and hence we were unable to distinguish between lean and fat mass. Although the sensitive analysis with adjusting for skinfold thickness has confirmed the main results, future studies should include precise measurements such as dual-energy X-ray absorptiometry to study the association between relative HS (calculated by fat mass) and hypertension.

## Conclusion

The relative HS level decreased during the last 15 years in urban and rural areas across different sex- and age-groups of the Chinese adult population. Lower relative HS was associated with a higher risk of hypertension. Considering the relative strength reflected the overall strength level, the findings provided evidence for the association between muscle strength and hypertension in large-scaled population.

## Supporting information

S1 Table The medians and interquartile of relative HS (HS to weight ratio) in four survey years. (DOCX)

S2 Table The medians and interquartile of relative HS (HS to weight ratio) in rural or urban areas in four survey years. (DOCX)

S3 Table The prevalence of hypertension in different tertile groups of relative HS(HS to weight ratio) in four survey years. (DOCX)

S4 Table The prevalence of hypertension in different tertile groups of relative HS (HS to weight ratio) across different age groups in 2014. (DOCX)

S5 Table Sensitivity analysis of relative HS (HS to weight ratio, category variable) in participants in the model adjusted for the skinfold thickness. (DOCX)

S6 Table Sensitive analysis of the association between another relative HS index (HS to BMI ratio, continuous variable) and hypertension (per IQR decrease). (DOCX)

S7 Table Sensitive analysis of the association between another relative HS index (handgrip strength to BMI ratio, category variable) and hypertension. (DOCX)

S8 Table Sensitive analysis of the associations between relative HS (category variable) and hypertension among different BMI groups from year 2005 to year 2014. (DOCX)

S9 Table Sensitive analysis of the associations between relative HS (category variable) and hypertension in male. (DOCX)

S10 Table Sensitive analysis of the associations between relative HS (category variable) and hypertension in female. (DOCX)

S11 Table Sensitive analysis of the associations between relative HS (category variable) and hypertension among different age groups. (DOCX)
