# Supplementary material for: Association between relative handgrip strength and hypertension in Chinese adults: An analysis of four successive national surveys with 712,442 individuals (2000-2014)
Source: PLoS One. 2021 Oct 28;16(10):e0258763. doi: 10.1371/journal.pone.0258763 (PMC8553048; doi:10.1371/journal.pone.0258763)
Supplement: S1 Table — (DOCX) [file pone.0258763.s001.docx]

Table S1 The Medians and interquartile of relative HS (HS to weight ratio) in four survey years.

|  | 2000 | | 2005 | | 2010 | | 2014 | |  |
| --- | --- | --- | --- | --- | --- | --- | --- | --- | --- |
|  | Median | IQR | Median | IQR | Median | IQR | Median | IQR | *p* Trend |
| Male | | | | | | | | |  |
| 20-24 | 0.753 | 0.166 | 0.728 | 0.167 | 0.710 | 0.173 | 0.677 | 0.174 | <0.05 |
| 25-29 | 0.743 | 0.174 | 0.718 | 0.168 | 0.681 | 0.167 | 0.651 | 0.168 | <0.05 |
| 30-34 | 0.726 | 0.169 | 0.709 | 0.166 | 0.671 | 0.161 | 0.641 | 0.162 | <0.05 |
| 35-39 | 0.711 | 0.170 | 0.696 | 0.164 | 0.670 | 0.160 | 0.640 | 0.155 | <0.05 |
| 40-44 | 0.695 | 0.165 | 0.678 | 0.156 | 0.656 | 0.155 | 0.639 | 0.152 | <0.05 |
| 45-49 | 0.678 | 0.159 | 0.662 | 0.154 | 0.641 | 0.154 | 0.619 | 0.152 | <0.05 |
| 50-54 | 0.650 | 0.159 | 0.645 | 0.159 | 0.623 | 0.153 | 0.602 | 0.154 | <0.05 |
| 55-59 | 0.628 | 0.159 | 0.620 | 0.157 | 0.602 | 0.152 | 0.586 | 0.157 | <0.05 |
| 50-64 | 0.589 | 0.165 | 0.583 | 0.162 | 0.568 | 0.158 | 0.553 | 0.161 | <0.05 |
| 65-69 | 0.554 | 0.160 | 0.551 | 0.158 | 0.537 | 0.152 | 0.530 | 0.158 | <0.05 |
| *p* Trend | <0.001 | | <0.001 | | <0.001 | | <0.001 | |  |
| Female | | | | | | | | |  |
| 20-24 | 0.537 | 0.135 | 0.524 | 0.132 | 0.504 | 0.130 | 0.488 | 0.135 | <0.05 |
| 25-29 | 0.535 | 0.136 | 0.523 | 0.132 | 0.495 | 0.133 | 0.475 | 0.133 | <0.05 |
| 30-34 | 0.532 | 0.132 | 0.521 | 0.132 | 0.496 | 0.127 | 0.472 | 0.130 | <0.05 |
| 35-39 | 0.518 | 0.133 | 0.510 | 0.134 | 0.489 | 0.129 | 0.471 | 0.128 | <0.05 |
| 40-44 | 0.503 | 0.134 | 0.491 | 0.131 | 0.473 | 0.129 | 0.460 | 0.127 | <0.05 |
| 45-49 | 0.480 | 0.132 | 0.468 | 0.129 | 0.453 | 0.125 | 0.443 | 0.125 | <0.05 |
| 50-54 | 0.458 | 0.130 | 0.441 | 0.125 | 0.428 | 0.125 | 0.421 | 0.121 | <0.05 |
| 55-59 | 0.439 | 0.127 | 0.427 | 0.123 | 0.412 | 0.124 | 0.411 | 0.125 | <0.05 |
| 50-64 | 0.414 | 0.125 | 0.406 | 0.124 | 0.395 | 0.116 | 0.387 | 0.119 | <0.05 |
| 65-69 | 0.401 | 0.129 | 0.395 | 0.119 | 0.380 | 0.121 | 0.373 | 0.118 | <0.05 |
| *p* Trend | <0.001 | | <0.001 | | <0.001 | | <0.001 | |  |

Notes: HS=handgrip strength; IQR=interquartile.
