# Supplementary material for: Association between relative handgrip strength and hypertension in Chinese adults: An analysis of four successive national surveys with 712,442 individuals (2000-2014)
Source: PLoS One. 2021 Oct 28;16(10):e0258763. doi: 10.1371/journal.pone.0258763 (PMC8553048; doi:10.1371/journal.pone.0258763)
Supplement: S2 Table — (DOCX) [file pone.0258763.s002.docx]

TableS2  The Medians and interquartile of relative HS (HS to weight ratio) in rural or urban areas in four survey years.

|  | 2000 | | 2005 | | 2010 | | 2014 | |  |
| --- | --- | --- | --- | --- | --- | --- | --- | --- | --- |
|  | Median | IQR | Median | IQR | Median | IQR | Median | IQR | *p* Trend |
| Male | | | | | | | | |  |
| rural | 0.686 | 0.195 | 0.671 | 0.187 | 0.640 | 0.182 | 0.619 | 0.179 | <0.001 |
| urban | 0.678 | 0.178 | 0.665 | 0.171 | 0.643 | 0.165 | 0.620 | 0.162 | <0.001 |
| *p* value | *p*<0.001 | | *p*<0.001 | | *p*<0.001 | | *p=*0.079 | |  |
| Female | | | | | | | | |  |
| rural | 0.488 | 0.160 | 0.468 | 0.157 | 0.447 | 0.152 | 0.438 | 0.145 | <0.001 |
| urban | 0.489 | 0.144 | 0.480 | 0.139 | 0.462 | 0.134 | 0.450 | 0.132 | <0.001 |
| *p* value | *p*=0.828 | | *p*<0.001 | | *p*<0.001 | | *p*<0.001 | |  |

Notes: HS=handgrip strength; IQR=interquartile.
