# Supplementary material for: Association between relative handgrip strength and hypertension in Chinese adults: An analysis of four successive national surveys with 712,442 individuals (2000-2014)
Source: PLoS One. 2021 Oct 28;16(10):e0258763. doi: 10.1371/journal.pone.0258763 (PMC8553048; doi:10.1371/journal.pone.0258763)
Supplement: S3 Table — (DOCX) [file pone.0258763.s003.docx]

Table S3 The prevalence of hypertension in different tertile groups of relative HS (HS to weight ratio) in four survey years

|  | 2000 | 2005 | 2010 | 2014 | *p* value |
| --- | --- | --- | --- | --- | --- |
| Total | | | | |  |
| Low HS | 24.59 (25.24-24.94) | 23.23 (22.9-23.56) | 25.61 (25.26-25.96) | 22.63 (22.28-22.98) | *p*<0.001 |
| Middle HS | 20.50 (20.18-20.82) | 18.28 (17.98-18.57) | 20.81 (20.49-21.13) | 18.69 (18.37-19.01) | *p*<0.001 |
| High HS | 16.60 (16.30-16.90) | 15.12 (14.84-15.40) | 16.99 (16.69-17.3) | 15.59 (15.28-15.89) | *p*<0.001 |
| *p* value | *p*<0.001 | *p*<0.001 | *p*<0.001 | *p*<0.001 |  |
| Male | | | | |  |
| Low HS | 28.76 (28.25-29.28) ^*^ | 28.67 (28.16-29.17) ^*^ | 31.93 (31.40-32.45) ^*^ | 29.50 (28.96-30.04) ^*^ | *p*<0.001 |
| Middle HS | 24.17 (23.69-24.65) ^*^ | 22.46 (22.00-22.92) ^*^ | 26.07 (25.58-26.56) ^*^ | 24.13 (23.63-24.63) ^*^ | *p*<0.001 |
| High HS | 18.80 (18.35-19.24) ^*^ | 17.85 (17.42-18.27) ^*^ | 20.27 (19.81-20.73) ^*^ | 19.35 (18.88-19.82) ^*^ | *p*<0.001 |
| *p* value | *p*<0.001 | *p*<0.001 | *p*<0.001 | *p*<0.001 |  |
| Female | | | | |  |
| Low HS | 20.36 (19.90-20.83) ^*^ | 17.82 (17.40-18.25) ^*^ | 19.23 (18.78-19.68) ^*^ | 15.77 (15.34-16.20) ^*^ | *p*<0.001 |
| Middle HS | 16.78 (16.36-17.21) ^*^ | 14.12 (13.73-14.50) ^*^ | 15.50 (15.09-15.91) ^*^ | 13.27 (12.87-13.66) ^*^ | *p*<0.001 |
| High HS | 14.37 (13.97-14.77) ^*^ | 12.40 (12.04-12.77) ^*^ | 13.69 (13.29-14.08) ^*^ | 11.83 (11.45-12.21) ^*^ | *p*<0.001 |
| *p* value | *p*<0.001 | *p*<0.001 | *p*<0.001 | *p*<0.001 |  |

Notes: HS=handgrip strength; *represent *p*<0.05 between men and women across different relative HS groups in different survey years.
