# Supplementary material for: Association between relative handgrip strength and hypertension in Chinese adults: An analysis of four successive national surveys with 712,442 individuals (2000-2014)
Source: PLoS One. 2021 Oct 28;16(10):e0258763. doi: 10.1371/journal.pone.0258763 (PMC8553048; doi:10.1371/journal.pone.0258763)
Supplement: S4 Table — (DOCX) [file pone.0258763.s004.docx]

Table S4 The prevalence of hypertension in different tertile groups of relative HS (HS to weight ratio) across different age groups in 2014

|  | Total,% | | | Male, % | | | Female,% | | |
| --- | --- | --- | --- | --- | --- | --- | --- | --- | --- |
| age group | low | middle | high | low | middle | high | low | middle | high |
| 20-24 | 9.12 (8.41-9.84) | 6.29 (5.69-6.89) | 5.21 (4.65-5.76) | 15.05 (13.8-16.31) | 9.83 (8.8-10.86) | 8.12 (7.16-9.09) | 3.15(2.53-3.77) | 2.72(2.15-3.28) | 2.27(1.75-2.8) |
| *p* | *p*<0.001 | | | *p*<0.001 | | | *p*=0.106 | | |
| 25-29 | 11.52 (10.73-12.32) | 7.66 (7.01-8.32) | 5.83 (5.25-6.41) | 19.64 (18.24-21.04) | 13.28 (12.1-14.46) | 9.61 (8.57-10.64) | 3.37(2.73-4.01) | 2.04(1.55-2.53) | 2.04(1.54-2.54) |
| *p* | *p* <0.001 | | | *p* <0.001 | | | *p*<0.001 | | |
| 30-34 | 9.35 (8.63-10.06) | 13.82 (12.95-14.68) | 7.27 (6.62-7.91) | 22.02(20.55-23.48) | 15.48(14.22-16.74) | 11.3 (10.18-12.42) | 5.71(4.89-6.52) | 3.28(2.67-3.9) | 3.29(2.66-3.91) |
| *p* | *p* <0.001 | | | *p* <0.001 | | | *p* <0.001 | | |
| 35-39 | 16.56 (15.63-17.5) | 13.66 (12.81-14.51) | 10.12 (9.36-10.88) | 24.78(23.23-26.32) | 21.07(19.63-22.5) | 14.78(13.52-16.05) | 8.5(7.52-9.49) | 6.41(5.56-7.27) | 5.54(4.73-6.35) |
| *p* | *p* <0.001 | | | *p* <0.001 | | | *p* <0.001 | | |
| 40-44 | 21.49 (20.43-22.55) | 16.88 (15.93-17.84) | 13.82 (12.93-14.71) | 29.7(28.03-31.37) | 23.74(22.21-25.27) | 18.27(16.86-19.68) | 13.17(11.93-14.41) | 9.92(8.84-11) | 9.3(8.24-10.37) |
| *p* | *p* <0.001 | | | *p* <0.001 | | | *p* <0.001 | | |
| 45-49 | 26.74 (25.58-27.89) | 21.99 (20.93-23.05) | 17.76 (16.76-18.76) | 35.08(33.32-36.84) | 28.89(27.24-30.53) | 21.77(20.25-23.3) | 18.37 (16.94-19.8) | 15.07(13.77-16.37) | 13.73(12.46-15) |
| *p* | *p* <0.001 | | | *p* <0.001 | | | *p* <0.001 | | |
| 50-54 | 29.94 (28.74-31.14) | 25.92 (24.78-27.05) | 22.13 (21.04-23.22) | 37.2(35.4-39) | 32.11(30.4-33.82) | 26.91(25.26-28.56) | 22.77(21.22-24.32) | 19.81(18.36-21.26) | 17.41(16.01-18.81) |
| *p* | *p* <0.001 | | | *p* <0.001 | | | *p* <0.001 | | |
| 55-59 | 32.75 (31.5-34) | 27.5 (26.32-28.67) | 24.09 (22.95-25.23) | 38.37(36.53-40.21) | 32.34(30.59-34.09) | 27.69(26-29.39) | 27.22 (25.54-28.89) | 22.74 (21.19-24.29) | 20.54 (19.03-22.06) |
| *p* | *p* <0.001 | | | *p* <0.001 | | | *p* <0.001 | | |
| 50-64 | 36.84 (35.35-38.33) | 34.35 (32.91-35.79) | 29.17 (27.77-30.58) | 40.85 (38.71-42.99) | 37.91 (35.83-39.99) | 32.16 (30.13-34.2) | 32.82 (30.78-34.87) | 30.78 (28.8-32.76) | 26.19 (24.27-28.1) |
| *p* | *p* <0.001 | | | *p* <0.001 | | | *p* <0.001 | | |
| 65-69 | 41.94 (40.38-43.51) | 37.92 (36.4-39.43) | 33.82 (32.32-35.32) | 44.13 (41.91-46.35) | 39.35 (37.2-41.5) | 35.24 (33.1-37.37) | 39.73 (37.52-41.93) | 36.46 (34.32-38.59) | 32.38 (30.27-34.49) |
| *p* | *p* <0.001 | | | *p* <0.001 | | | *p* <0.001 | | |

Notes: data are n (%); HS=handgrip strength.
