# Supplementary material for: Association between relative handgrip strength and hypertension in Chinese adults: An analysis of four successive national surveys with 712,442 individuals (2000-2014)
Source: PLoS One. 2021 Oct 28;16(10):e0258763. doi: 10.1371/journal.pone.0258763 (PMC8553048; doi:10.1371/journal.pone.0258763)
Supplement: S5 Table — (DOCX) [file pone.0258763.s005.docx]

Table S5 Sensitivity analysis of relative HS (HS to weight ratio, category variable) in participants in the model adjusted for the skinfold thickness.

|  | High HS | Middle HS | | Low HS | |
| --- | --- | --- | --- | --- | --- |
|  |  | OR (95% CI) | *p* | OR (95% CI) | *p* |
| 2000 | | | | |  |
| Model 3 | REF | 1.16(1.12-1.20) | ＜0.001 | 1.31(1.27-1.35) | ＜0.001 |
| 2005 | | | | |  |
| Model 3 | REF | 1.11 (1.07-1.15) | ＜0.001 | 1.34 (1.30-1.38) | ＜0.001 |
| 2010 | | | | |  |
| Model 3 | REF | 1.10 (1.07-1.14) | ＜0.001 | 1.26 (1.22-1.30) | ＜0.001 |
| 2014 | | | | |  |
| Model 3 | REF | 1.11 (1.07-1.15) | ＜0.001 | 1.26 (1.22-1.31) | ＜0.001 |

Notes: HS=handgrip strength; OR=odds ratios; CI=confidence interval; REF=reference group.

Model 3: adjusted for age, sex, region (urban or rural), inner-province economic status (high, middle, low), nationality, education level, career, exercise (at least 60 mins/week or not) and skinfold thickness.
