# Supplementary material for: Association between relative handgrip strength and hypertension in Chinese adults: An analysis of four successive national surveys with 712,442 individuals (2000-2014)
Source: PLoS One. 2021 Oct 28;16(10):e0258763. doi: 10.1371/journal.pone.0258763 (PMC8553048; doi:10.1371/journal.pone.0258763)
Supplement: S6 Table — (DOCX) [file pone.0258763.s006.docx]

Table S6 Sensitive Analysis of the association between another relative HS index (HS to BMI ratio, continuous variable) and hypertension (per IQR decrease).

| Survey year　 Model | OR (95% CI) | *p* |
| --- | --- | --- |
| 2000 |  |  |
| Crude | 1.23(1.21-1.24) | ＜0.001 |
| Model 1 | 1.26(1.24-1.29) | ＜0.001 |
| Model 2 | 1.26(1.24-1.29) | ＜0.001 |
| Model 3 | 1.12(1.10-1.15) | ＜0.001 |
| 2005 |  |  |
| Crude | 1.17(1.16-1.18) | ＜0.001 |
| Model 1 | 1.36(1.33-1.39) | ＜0.001 |
| Model 2 | 1.35(1.32-1.38) | ＜0.001 |
| Model 3 | 1.16(1.14-1.19) | ＜0.001 |
| 2010 |  |  |
| Crude | 1.17(1.15-1.19) | ＜0.001 |
| Model 1 | 1.53(1.49-1.58) | ＜0.001 |
| Model 2 | 1.50(1.46-1.54) | ＜0.001 |
| Model 3 | 1.18(1.14-1.21) | ＜0.001 |
| 2014 |  |  |
| Crude | 1.07(1.05-1.09) | ＜0.001 |
| Model 1 | 1.46(1.41-1.50) | ＜0.001 |
| Model 2 | 1.44(1.40-1.48) | ＜0.001 |
| Model 3 | 1.18(1.15-1.22) | ＜0.001 |

Notes: HS=handgrip strength; IQR=interquartile; OR=odds ratio; CI=confidence interval; REF=reference group.

Crude Model: with the province of each participant was used as the random effect.

Model 1: adjusted for age and sex.

Model 2: adjusted for age, sex, region (urban or rural), inner-province economic status (high, middle, low), nationality, education level, career, exercise (at least 60 mins/week or not).

Model 3: adjusted for age, sex, region (urban or rural), inner-province economic status (high, middle, low), nationality, education level, career, exercise (at least 60 mins/week or not) and skinfold thickness.
