# Supplementary material for: Association between relative handgrip strength and hypertension in Chinese adults: An analysis of four successive national surveys with 712,442 individuals (2000-2014)
Source: PLoS One. 2021 Oct 28;16(10):e0258763. doi: 10.1371/journal.pone.0258763 (PMC8553048; doi:10.1371/journal.pone.0258763)
Supplement: S7 Table — (DOCX) [file pone.0258763.s007.docx]

S7 Table Sensitive Analysis of the association between another relative HS index (handgrip strength to BMI ratio, category variable) and hypertension

|  | High HS | Middle HS | | Low HS | |
| --- | --- | --- | --- | --- | --- |
|  |  | OR (95% CI) | *p* | OR (95% CI) | *p* |
| 2000 | | | | | |
| Crude | REF | 1.23 (1.20-1.27) | ＜0.001 | 1.47 (1.42-1.51) | ＜0.001 |
| Model 1 | REF | 1.25 (1.21-1.29) | ＜0.001 | 1.51 (1.46-1.56) | ＜0.001 |
| Model 2 | REF | 1.26 (1.22-1.30) | ＜0.001 | 1.51 (1.47-1.56) | ＜0.001 |
| Model 3 | REF | 1.14 (1.10-1.17) | ＜0.001 | 1.26 (1.22-1.30) | ＜0.001 |
| 2005 | | | | | |
| Crude | REF | 1.31 (1.27-1.35) | ＜0.001 | 1.66 (1.61-1.71) | ＜0.001 |
| Model 1 | REF | 1.30 (1.26-1.35) | ＜0.001 | 1.68 (1.63-1.74) | ＜0.001 |
| Model 2 | REF | 1.30 (1.26-1.35) | ＜0.001 | 1.66 (1.61-1.72) | ＜0.001 |
| Model 3 | REF | 1.16 (1.12-1.20) | ＜0.001 | 1.33 (1.29-1.37) | ＜0.001 |
| 2010 | | | | | |
| Crude | REF | 1.24 (1.21-1.28) | ＜0.001 | 1.55 (1.51-1.59) | ＜0.001 |
| Model 1 | REF | 1.26 (1.22-1.30) | ＜0.001 | 1.60 (1.56-1.65) | ＜0.001 |
| Model 2 | REF | 1.25 (1.22-1.29) | ＜0.001 | 1.57 (1.52-1.62) | ＜0.001 |
| Model 3 | REF | 1.10 (1.06-1.13) | ＜0.001 | 1.23 (1.19-1.27) | ＜0.001 |
| 2014 | | | | | |
| Crude | REF | 1.22 (1.18-1.26) | ＜0.001 | 1.50 (1.45-1.54) | ＜0.001 |
| Model 1 | REF | 1.23 (1.19-1.28) | ＜0.001 | 1.54 (1.49-1.59) | ＜0.001 |
| Model 2 | REF | 1.24 (1.20-1.28) | ＜0.001 | 1.52 (1.47-1.57) | ＜0.001 |
| Model 3 | REF | 1.12 (1.08-1.16) | ＜0.001 | 1.25 (1.20-1.29) | ＜0.001 |

Notes: HS=handgrip strength; OR=odds ratio; CI=confidence interval; REF=reference group; BMI=body mass index.

Crude Model: with the province of each participant was used as the random effect.

Model 1: adjusted for age and sex.

Model 2: adjusted for age, sex, region (urban or rural), inner-province economic status (high, middle, low), nationality, education level, career, exercise (at least 60 mins/week or not).

Model 3: adjusted for age, sex, region (urban or rural), inner-province economic status (high, middle, low), nationality, education level, career, exercise (at least 60 mins/week or not) and skinfold thickness.
