# Supplementary material for: Association between relative handgrip strength and hypertension in Chinese adults: An analysis of four successive national surveys with 712,442 individuals (2000-2014)
Source: PLoS One. 2021 Oct 28;16(10):e0258763. doi: 10.1371/journal.pone.0258763 (PMC8553048; doi:10.1371/journal.pone.0258763)
Supplement: S8 Table — (DOCX) [file pone.0258763.s008.docx]

Table S8 Sensitive Analysis of the associations between relative HS (category variable) and hypertension among different BMI groups from year 2005 to year 2014.

|  | High HS | Middle HS | | Low HS | |
| --- | --- | --- | --- | --- | --- |
|  |  | OR (95% CI) | *p* | OR (95% CI) | *p* |
| **Wasting (n=23,970)** | | | | | |
| Crude | REF | 0.74 (0.65-0.84) | *p*<0.001 | 0.93 (0.79-1.09) | 0.37 |
| Model 1 | REF | 0.88 (0.76-1.00) | 0.05 | 1.09 (0.91-1.30) | 0.04 |
| Model 2 | REF | 0.89 (0.77-1.02) | 0.08 | 1.08 (0.90-1.29) | 0.41 |
| **Normal (n=285,455)** | | | | | |
| Crude | REF | 0.93 (0.91-0.96) | *p*<0.001 | 0.95 (0.93-0.98) | 0.001 |
| Model 1 | REF | 1.03 (1.00-1.05) | 0.06 | 1.09 (1.06-1.12) | *p*<0.001 |
| Model 2 | REF | 1.04 (1.01-1.07) | *p*<0.001 | 1.08 (1.04-1.12) | *p*<0.001 |
| **Overweight and obesity (n=224,903)** | | | | | |
| Crude | REF | 0.99 (0.97-1.02) | 0.59 | 1.07 (1.04-1.10) | *p*<0.001 |
| Model 1 | REF | 1.05 (1.02-1.08) | 0.002 | 1.22 (1.19-1.26) | *p*<0.001 |
| Model 2 | REF | 1.05 (1.02-1.08) | *p*<0.001 | 1.21 (1.18-1.24) | *p*<0.001 |

Notes: HS=handgrip strength; OR=odds ratio; CI=confidence interval; REF=reference group.

Crude Model: adjust survey year and the province of each participant was used as the random effect.

Model 1: adjusted for age and sex, survey year

Model 2: adjusted for age, sex, survey year, region (urban or rural), inner-province economic status (high, middle, low), nationality, education level, career, exercise (at least 60 mins/week or not).
