# Supplementary material for: Association between relative handgrip strength and hypertension in Chinese adults: An analysis of four successive national surveys with 712,442 individuals (2000-2014)
Source: PLoS One. 2021 Oct 28;16(10):e0258763. doi: 10.1371/journal.pone.0258763 (PMC8553048; doi:10.1371/journal.pone.0258763)
Supplement: S9 Table — (DOCX) [file pone.0258763.s009.docx]

Table S9 Sensitive Analysis of the associations between relative HS (category variable) and hypertension in Male.

|  | High HS | Middle HS | | Low HS | |
| --- | --- | --- | --- | --- | --- |
|  |  | OR (95% CI) | *p* | OR (95% CI) | *p* |
| 2000 | | | | | |
| Crude | REF | 1.34 (1.29-1.40) | ＜0.001 | 1.65 (1.58-1.71) | ＜0.001 |
| Model 1 | REF | 1.36 (1.31-1.42) | ＜0.001 | 1.69 (1.62-1.76) | ＜0.001 |
| Model 2 | REF | 1.39 (1.33-1.45) | ＜0.001 | 1.72 (1.64-1.79) | ＜0.001 |
| 2005 | | | | | |
| Crude | REF | 1.32 (1.27-1.37) | ＜0.001 | 1.80 (1.73-1.87) | ＜0.001 |
| Model 1 | REF | 1.34 (1.29-1.40) | ＜0.001 | 1.87 (1.80-1.95) | ＜0.001 |
| Model 2 | REF | 1.35 (1.30-1.41) | ＜0.001 | 1.89 (1.81-1.97) | ＜0.001 |
| 2010 | | | | | |
| Crude | REF | 1.35 (1.30-1.40) | ＜0.001 | 1.74 (1.68-1.81) | ＜0.001 |
| Model 1 | REF | 1.37 (1.32-1.42) | ＜0.001 | 1.79 (1.72-1.86) | ＜0.001 |
| Model 2 | REF | 1.38 (1.32-1.43) | ＜0.001 | 1.80 (1.73-1.87) | ＜0.001 |
| 2014 | | | | | |
| Crude | REF | 1.31 (1.25-1.36) | ＜0.001 | 1.68 (1.61-1.75) | ＜0.001 |
| Model 1 | REF | 1.32 (1.26-1.37) | ＜0.001 | 1.71 (1.64-1.79) | ＜0.001 |
| Model 2 | REF | 1.34 (1.28-1.39) | ＜0.001 | 1.75 (1.67-1.82) | ＜0.001 |

Notes: HS=handgrip strength. Crude Model: with the province of each participant was used as the random effect.

Model 1: adjusted for age. Model 2: adjusted for age, region (urban or rural), inner-province economic status (high, middle, low), nationality, education level, career, exercise (at least 60 mins/week or not).
