# Supplementary material for: Association between relative handgrip strength and hypertension in Chinese adults: An analysis of four successive national surveys with 712,442 individuals (2000-2014)
Source: PLoS One. 2021 Oct 28;16(10):e0258763. doi: 10.1371/journal.pone.0258763 (PMC8553048; doi:10.1371/journal.pone.0258763)
Supplement: S10 Table — (DOCX) [file pone.0258763.s010.docx]

Table S10  Sensitive Analysis of the associations between relative HS (category variable) and hypertension in Female.

|  | High HS | Middle HS | | Low HS | |
| --- | --- | --- | --- | --- | --- |
|  |  | OR (95% CI) | *p* | OR (95% CI) | *p* |
| 2000 | | | | | |
| Crude | REF | 1.16 (1.11-1.22) | ＜0.001 | 1.41 (1.35-1.47) | ＜0.001 |
| Model 1 | REF | 1.18 (1.12-1.24) | ＜0.001 | 1.45 (1.39-1.53) | ＜0.001 |
| Model 2 | REF | 1.20 (1.15-1.27) | ＜0.001 | 1.47 (1.40-1.55) | ＜0.001 |
| 2005 | | | | | |
| Crude | REF | 1.13 (1.08-1.19) | ＜0.001 | 1.47 (1.41.-1.54) | ＜0.001 |
| Model 1 | REF | 1.14 (1.08-1.20) | ＜0.001 | 1.52 (1.45-1.60) | ＜0.001 |
| Model 2 | REF | 1.15 (1.09-1.21) | ＜0.001 | 1.48 (1.41-1.56) | ＜0.001 |
| 2010 | | | | | |
| Crude | REF | 1.14 (1.09-1.19) | ＜0.001 | 1.45 (1.38-1.51) | ＜0.001 |
| Model 1 | REF | 1.15 (1.10-1.21) | ＜0.001 | 1.50 (1.43-1.57) | ＜0.001 |
| Model 2 | REF | 1.14 (1.09-1.20) | ＜0.001 | 1.43 (1.37-1.51) | ＜0.001 |
| 2014 | | | | | |
| Crude | REF | 1.13 (1.08-1.19) | ＜0.001 | 1.37 (1.30-1.44) | ＜0.001 |
| Model 1 | REF | 1.14 (1.08-1.20) | ＜0.001 | 1.40 (1.33-1.47) | ＜0.001 |
| Model 2 | REF | 1.14 (1.08-1.20) | ＜0.001 | 1.35 (1.28-1.43) | ＜0.001 |

Notes: HS=handgrip strength. Crude Model: with the province of each participant was used as the random effect.

Model 1: adjusted for age. Model 2: adjusted for age, region (urban or rural), inner-province economic status (high, middle, low), nationality, education level, career, exercise (at least 60 mins/week or not).
