# Supplementary material for: Association between relative handgrip strength and hypertension in Chinese adults: An analysis of four successive national surveys with 712,442 individuals (2000-2014)
Source: PLoS One. 2021 Oct 28;16(10):e0258763. doi: 10.1371/journal.pone.0258763 (PMC8553048; doi:10.1371/journal.pone.0258763)
Supplement: S11 Table — (DOCX) [file pone.0258763.s011.docx]

S11 Table Sensitive Analysis of the associations between relative HS (category variable) and hypertension among different age groups.

|  | High HS | Middle HS | | Low HS | |
| --- | --- | --- | --- | --- | --- |
|  |  | OR (95% CI) | *p* | OR (95% CI) | *p* |
| 20-29 age group | | | | | |
| Crude | REF | 1.27 (1.14-1.40) | ＜0.001 | 1.91 (1.73-2.11) | ＜0.001 |
| Model 1 | REF | 1.27 (1.15-1.42) | ＜0.001 | 1.95 (1.76-2.15) | ＜0.001 |
| Model 2 | REF | 1.31 (1.18-1.46) | ＜0.001 | 2.03 (1.83-2.24) | ＜0.001 |
| 30-39 age group | | | | | |
| Crude | REF | 1.34 (1.23-1.46) | ＜0.001 | 1.82 (1.68-1.97) | ＜0.001 |
| Model 1 | REF | 1.36 (1.25-1.48) | ＜0.001 | 1.86 (1.71-2.02) | ＜0.001 |
| Model 2 | REF | 1.38 (1.27-1.51) | ＜0.001 | 1.92 (1.77-2.09) | ＜0.001 |
| 40-49 age group | | | | | |
| Crude | REF | 1.27 (1.18-1.36) | ＜0.001 | 1.61 (1.50-1.72) | ＜0.001 |
| Model 1 | REF | 1.27 (1.19-1.37) | ＜0.001 | 1.62 (1.52-1.74) | ＜0.001 |
| Model 2 | REF | 1.30 (1.21-1.39) | ＜0.001 | 1.62 (1.51-1.73) | ＜0.001 |
| 50-59 age group | | | | | |
| Crude | REF | 1.18 (1.11-1.25) | ＜0.001 | 1.44 (1.35-1.53) | ＜0.001 |
| Model 1 | REF | 1.18 (1.11-1.26) | ＜0.001 | 1.44 (1.36-1.54) | ＜0.001 |
| Model 2 | REF | 1.19 (1.12-1.27) | ＜0.001 | 1.44 (1.35-1.53) | ＜0.001 |
| 60-69 age group | | | | | |
| Crude | REF | 1.22 (1.14-1.31) | ＜0.001 | 1.37 (1.28-1.47) | ＜0.001 |
| Model 1 | REF | 1.22 (1.14-1.31) | ＜0.001 | 1.37 (1.28-1.47) | ＜0.001 |
| Model 2 | REF | 1.23 (1.15-1.31) | ＜0.001 | 1.34 (1.25-1.44) | ＜0.001 |

Notes: HS=handgrip strength. Crude Model: with the province of each participant was used as the random effect.

Model 1: adjusted for sex. Model 2: adjusted for sex, region (urban or rural), inner-province economic status (high, middle, low), nationality, education level, career, exercise (at least 60 mins/week or not).
